# The Lancet in Dentition

**Published:** 1882-09

**Authors:** Thos. S. Sozinskey


### ARTICLE II.

### *The Lancet in Dentition.*

BY THOS. S. SOZINSKEY, M. D., PH. D.,

It is noted by Southy, in his common-place book, "The Doctor," that "Rogers, who had been about the person of Charles the Second, died at ninety-six, in cutting his teeth.

He had four, and many more were coming, which so inflamed his gums that it proved fatal." Poor old Rrogers!

"Cutting of the teeth" is, rightly or wrongly, credited popularly, with the destruction of many lives, and it would seem from this case that it is liable to be accused of being at work even in the terminal stage of existence. In the nursery it has always, perhaps, been regarded as a very serious matter. Nevertheless, this common belief, like many another, may not be well founded. Cumulative tradition has possibly served to, at least excessively emphasize the balefulness of the process. It may, however, be taken for granted, I believe, that the amount of suffering experienced by children in general while getting their primary teeth is very considerable. It causes decided uneasiness in almost all, and decided danger in some. If it rarely or never destroys life directly, it certainly not unfrequently does indirectly. But I am not sure that it is entirely wrong to give it as the actual cause of death in not a few cases. This is at any rate done. In Philadelphia, about thirty deaths are attributed to it, on the average, yearly, in the bills of mortality. During the census year ending June 30th, 1870, there were three thousand two hundred and forty-seven deaths attributed to it in the United States. There is assuredly some ground for the prevailing belief that dentition is the direct or indirect source from which flow many of the ills and a large share of the mortality of babies.

It would appear, however, that there are some physicians who seriously regard the coming of the teeth as an extremely innocent matter, doing, as they say, a purely physiological process. I would advise such that it is assuming a great deal to take it for granted that dentition is in all cases a purely physiological process. Perhaps in no child is it absolutely physiological; it is at best only approximately physiological. In a recent address, Dr. B. W. Richardson went so far as to declare that he "had never seen a healthy child," never one "that had not in it either some actual or latent constitutional disease." At any rate, there is good reason for believing that in a large proportion of children, especially in large cities, dentition is of decidedly pathological import. As is true of many other troubles, it generally tends to be more marked in proportion to the degree of unhealthiness of the child. And let it be borne in mind that the sensibility of all infants is great, and of some sickly ones such that even a trifling irritation may be followed by serious constitutional and other effects.

But it is not my purpose to speak of the subject of dentition. This has been done very well in a lecture published in this journal recently. It may be well, however, to say, in the words of John Hunter, in his admirable treatise on the natural history and diseases of the teeth, "the symptoms are so various in different children, and often in the same child, that it is difficult to conceive them to be from the same origin, and the varieties are such as seem to be beyond our knowledge."

Now, when to the discerning senses of the observing sensible physicians a child is suffering severely from the eruption of one or more teeth, relief should, of course be procured as soon as possible. How is this end to be attained? The advice given by many amounts to little more than to use a soothing-syrup—a la Winslow—of some sort. Meeting the various indications is, in truth, in numerous cases, far from being an easy matter. Such measures as serve to favor the general health are particularly indicated in every case. Careful regulation of the diet, daily bathing and a fair amount of exercise, especially in the open air, are among the items of great significance. Make the process as little pathological as possible by making the condition of the child as physiological as possible. Anodynes, antacids, febrifuges and other remedies, including local applications, may or may not be called for. But I believe the remedy most imperiously called for, in at least bad cases, lies in the use of the lancet.

Of the use of the lancet in dentition it is curious to observe how different are the opinions entertained. Some do not hesitate to say that it is of no value. Thus, in a recent widely circulated work on the diseases of children, by Dr. Henoch, a Berlin practitioner of seemingly large experience, it is said : "It is now generally held that every attempt to facilate the eruption of the teeth, and thus remove the symptoms due to difficult dentition, is absolutely useless. I have in earlier years performed sacrification with sufficient frequency to convince myself of its entire inutility ; and it even appears to me that the cicatrix formed may increase the difficulties connected with the penetration of the teeth." Here is what Dr. J. Lewis Smith says on the subject, in his work on the diseases of infancy and childhood : "The gum-lancet is now much less frequently employed than formerly. It is used more by the ignorant practitioner who is deficient in the ability to diagnosticate obscure diseases, than by one of intelligence, who can discern more clearly the true pathological state. Its use is more frequent in some countries, as in England, under the teaching of great names, than in others, as France where the highest authorities, as Rilliet and Barthez, discountenance it. . . . I know no accidents of dentition which require prompt scarification, except suppurative inflammation of the gums, convulsions, and paralysis. In other cases the operation may be safely postponed till other measures have been employed." In Drs. Meigs and Pepper's treatise on the diseases of children it is said : "Lancing of the gums is undoubtedly a most important point in this [laryngismus stridulus] and other diseases of childhood connected with dentition. We have long been convinced, however, from personal observation, that a resort to this operation merely because the child is passing through the period of dentition is at least useless. We have never found it to do any good unless the teeth are near enough to the surface to produce manifest swelling, attended with heat and soreness of the gums. So long as the gum is hard, insensible, not turgid, and of ts natural color, and the mouth not hot, cutting has done no good." That wise old master of the healing art, John Hunter, after speaking of symptoms arising from dentition says, "So far as my experience has taught me, to cut the gum down to the teeth appears to be the only method of cure."

Truly the authors quoted from differ sufficiently as to the value of the lancet in dentition. It is worth while to dwell a little on what they say.

Dr. Henoch speaks very rashly to say the least, in declaring that "it is generally held that every attempt to facilitate the eruption of the teeth is absolutely useless." The idea was not formed from an extensive acquaintance with the literature of the subject. Again, the statement of the Doctor as to his experience with the use of the lancet may well lead one to infer that he has had hardly any experience with it. He is like those who, although having no knowledge of the use of the lancet in general medicine, yet unhesitatingly venture to condemn it. What he has to say about "the cicatrix" may rightly produce the impression that he has not made himself master of the subject. If the lancing is properly done, there need be no cicatrix; and if there be one it will not serve to retard the "penetration" of the tooth. As Hunter long ago said, at the point of cicatrix the gum offers the least resistance to the appearance of the tooth.

Dr. Smith is doubtless right in holding that ignorant practitioners are very apt to use the lancet when not required, and in his intimation, that the use of it is largely a matter of fashion. But I am also quite sure that too many practitioners who are usually regarded as intelligent do not use it as often as they should. His statement that he "knows no accidents of dentition which require prompt scarification, except suppurative inflammation of the gums, covulsions and paralysis," is a trifle extraordinary. What of cases of, say, larynigismus stridulus and of cholera infantum, in which dentition bears largely a causative relation?

Except in the diseases he mentions, the Doctor would postpone the operation "till other measures have been employed." It scarcely follows from this that he is acquainted with the cardinal principle of sound medical practice. In treating of the same subject, Hunter correctly remarks, "It would be better at once to remove the cause, than to be attemping from time to time to remove or palliate the effect." Wasting time trying to palliate effects, while the cause is allowed to act, lets many a little innocent go unnecessarily and speedily into the grave.

Drs. Meigs and Pepper "have long been convinced, from personal observation, that a resort to the operation merely because the child is passing through the period of dentition is at least useless." It is rather strange that they were ever otherwise than 'convinced.' No sensible physician cuts a child's gums indiscriminately. Resorting to the lancet when there is no evident need for it, no matter how the child is affected, is, as Dr. West fitly says, "barbarous empiricism." The idea that it is the approach of the teeth "near to the surface" which produces the troubles which are met with in dentition is not of necessity a truth. At least the main source of trouble in the appearonce of a tooth is not at the surface; it is at the base. The backward pressure on the sensitive nerves and other parts give rise largely to the pain and irritation about the gum. This backward pressure may set up trouble a considerable time before the tooth nears the surface, the tension then being almost or quite as great as later. In fact it often, or perhaps I should say generally, happens that there is a spell of trouble some time before the tooth is about to make its appearance. In some it is very marked and it may be repeated. The capsule becomes distended and the surrounding parts hyperæmic and painful, and there is a free flow of saliva. In any case where there is evidently decided irritation about the gum the lancet should be used. It should be used even if for no other reason than to relieve the child of local pain, of toothache, an affliction which, as many know, and Burns told in expressive poetic phrase, is something awful enough in itself, a pain which at its worst Bacon had reason in believing to be "one of the sharpest of pains." There need be no hesitancy in using the lancet repeatedly if the gum is at any point tense and angry looking. Says Hunter, "I have performed the operation about ten times upon the same tooth, where the disease had recurred so often, and every time with the absolute removal of the symptoms."

That the lancet will afford relief in the presence of very marked symptoms every one surely is aware who knows how to use it. It is an instrument by which much may be done to lessen suffering, cure disease and save life. Here is a delicate child, somewhat over a year old, languid, pale, the victim of one or two convulsions, with contracted brows, a temperature of from 101° to 102°, a tendency to diarrhœa and a slight bronchitic cough. I lance his cutting tooth, and within a few hours he is almost entirely well. I have done the thing so often in his case that I know precisely what the result will be. This lymphatic child of fifteen months has a developing croup; the hurried breathing, the noise in the throat and all the other symptoms are becoming decided. I lance his appearing eye teeth and he begins immediately to get well. There is a strong looking boy, nearly two years old, with a temperature of 103°, and a marked cold on his chest. I prescribe appropriate remedies but at the end of two days he is no better. I lance a pair of coming molars, and next day there is no fever hardly, and the cough speedily vanishes. That poorly cared for child, who has about completed her first year, is evidently, from the high fever (103.5°), nausea and tendency to diarrhœa, drifting into the fatal grasp of cholera infantum, the midsummer heat favoring the development of that serious infantile disease. I look at her mouth and find that the gums over both the eye, and the stomach-teeth are tense and angry looking. I lance these points, and very simple measures restore the patient, within fortyeight hours, to her usual state of health. But I need not give an extended recital of cases. I am of the opinion that early, and, if need be, repeated lancing would save many who should otherwise perish from convulsions, bronchitis, cholera infantum and other diseases. Especially in spent children, in the warmer part of the year, should there be no delay. Statistics of the census year ending June 30th, 1870, show that there are more deaths attributable to dentition in the third quarter of the year than in the two preceding ones together, and many more than in the first and fourth together. The Philadelphia bills of mortality present a very similar showing.

In order that lancing the gums may do as much good as possible it must be done well, but, of course, not butcherly. A plain incision with a very sharp lancet is not enough; it will generally do only a little good. This is what is not uncommonly done. Let there be several more or less irregular incisions made, with a somewhat dull lancet, and the hoped for result will very certainly follow. In bad cases the gum should be very thoroughly scarified, so thoroughly that there will be little or no likelihood of the parts uniting over the tooth. And it is proper to extend the incisions well to the sides of the coming tooth. This may be called for after the tooth has partly appeared. Whatever bleeding occurs will be beneficial; it will serve to relieve the hyperæmia and pain. In fact, in not a few cases it is the bleeding that is principally called for; local depletion affords immediate relief. It will not be harmfully profuse, likely, in any case. Says Hunter, "I never saw a case where the bleeding proved either inconvenient or dangerous.

What harm may result from lancing the gums, under any but very extraordinary circumstances, it would be hard to state. I do not feel sure that it can do any, and in this belief I am fortified by the opinions of perhaps all dentists and others who have carefully studied the matter. It may cause temporary pain. Still, this is not very marked.

Many a child will open its mouth and have several teeth thoroughly lanced without giving a whimper. As for the tenderness of the gum, which there may be for a day or two, from thorough lancing, it is comparatively a small matter. The idea that lancing will retard the development of the tooth has, I believe, no just foundation. It would appear to be based on the erroneous notion that when lancing is practiced the tooth should immediately appear. It is probable that in no case is there any more than an apparent delay. As already stated, the irritation about the gum which calls for the use of the lancet may arise weeks before the tooth approaches the surface. A tooth does not receive its nourishment from the structures above or about its crown. The cicatrix and hemorrhage I have already spoken of at sufficient length. In all proper cases the lancet may, then, be used, with confidence that it is a markedly non-injurious, as well as an almost infallible means of relief.—*Med. and Surg. Reporter.*

---

